# Central venous hyperoxia is related to changes in tissue perfusion and morbi-mortality of patients in shock

**DOI:** 10.1186/cc10863

**Published:** 2012-03-20

**Authors:** G Friedman, A Do Canto, D Araujo

**Affiliations:** 1 UFRGS, Porto Alegre, Brazil; 2 Complexo Hospitalar Santa Casa, Porto Alegre, Brazil

## Introduction

Mixed or central venous hyperoxia is associated with organ dysfunction and worse mortality. Venous hyperoxia may reflect altered tissue oxygen extraction. We aim to assess the relationship between central venous hyperoxia (ScvO_2_) and markers of tissue perfusion; and to evaluate the relationship between central venous hyperoxia and morbidity.

## Methods

The setting was a university general ICU with 18 beds. The population was adult patients (age >18 years) in circulatory shock. Blood lactate, arterial and central venous blood gases were collected on admission to the study and after 6, 12, 18 and 24 hours of shock. Venous hyperoxia was defined as a ScvO_2 _≥85%. The severity of the patients was assessed using the APACHE II score on admission to the study. Mortality was evaluated in the ICU and after 28 days.

## Results

Preliminary data from 40 patients (205 measurements) are presented. Mean blood lactate levels were higher (3.2 vs. 2.3 mmol/l), the mean venoarterial CO_2 _difference was lower (4.7 vs. 5.8 mmHg), and the mean base deficit was greater (11 vs. 8 mEq/l) for patients with venous hyperoxia at any time. Mean APACHE II score was higher (28 vs. 24) for patients with venous hyperoxia. The ICU mortality was higher (4/5 (80%) vs. 17/35 (46%)) among patients who already had venous hyperoxia at time 0. The proportion of death remained the same in the next day among patients that persisted or developed venous hyperoxia in the following 18 hours.

## Conclusion

These preliminary data suggest that the presence of central venous hyperoxia is associated with persistent changes in perfusion. The presence of venous hyperoxia at both the onset of shock and in the following hours is associated with a worse clinical outcome.
